# Showing the Way: Oncolytic Adenoviruses as Chaperones of Immunostimulatory Adjuncts

**DOI:** 10.3390/biomedicines4030023

**Published:** 2016-09-19

**Authors:** Jing Li Huang, Christopher J. LaRocca, Masato Yamamoto

**Affiliations:** Department of Surgery, University of Minnesota, Minneapolis, MN 55455, USA; jingx014@umn.edu (J.L.H.); clarocca@umn.edu (C.J.L.)

**Keywords:** oncolytic adenovirus, immunotherapy, oncolytic virotherapy, GM-CSF (granulocyte macrophage colony-stimulating factor), interferon-α, interferon-γ, interleukin-12, CD40L, CTLA-4

## Abstract

Oncolytic adenoviruses (OAds) are increasingly recognized as vectors for immunotherapy in the treatment of various solid tumors. The myriads of advantages of using adenovirus include targeted specificity upon infection and selective replication, which lead to localized viral burst, exponential spread of OAds, and antitumor effect. OAds can also induce a strong immune reaction due to the massive release of tumor antigens upon cytolysis and the presence of viral antigens. This review will highlight recent advances in adenoviral vectors expressing immunostimulatory effectors, such as GM-CSF (granulocyte macrophage colony-stimulating factor), interferon-α, interleukin-12, and CD40L. We will also discuss the combination of OAds with other immunotherapeutic strategies and describe the current understanding of how adenoviral vectors interact with the immune system to eliminate cancer cells.

## 1. Introduction

Research over the past decade has brought forth a greater understanding of tumor immunity. Multiple cross-talk pathways between cancer and cells of the immune system mediate in the development of a tumor microenvironment where cancer cells can evade immune detection [1]. While infiltration of dendritic cells, macrophages, natural killer (NK) cells occurs early in tumor development [1,2], the pro-inflammatory actions of these cells are counteracted by immunosuppressive cells such as immature myeloid cells (the myeloid-derived suppressor cells, MDSC), regulatory T cells, and tumor-associated macrophages [3]. The tumor and its stroma produce factors that attract immature myeloid cells, which in turn produce cytokines such as tumor-growth factor-beta (TGF-β), interleukin-10 (IL-10), arginase I and myeloperoxidase that prevent myeloid cell maturation and decrease lymphocyte activation [4]. The production of these mediators likely leads to the development of M2-skewed tumor-associated macrophages, which enhance tumor proliferation, increase stromal deposition and stimulate angiogenesis and remodeling [5]. In addition, cytokines, such as TGF-β, can promote the differentiation of CD4+ T cells into regulatory T cells, which further suppress the activation of lymphocytes and produce TGF-β and IL-10. The immunosuppressive state of the tumor microenvironment is counteracted by the presence of Th1 immunity, which ultimately leads to the activation of effector T cells. The maturation of dendritic cells results in greater antigen presentation and increased IL-12 production, which tips the balance away from a Th2-type response. In turn, effector CD4+ and CD8+ T cells are stimulated in an antigen-directed manner to target tumor cells. Advances in the understanding of tumor immunity have allowed researchers to employ new strategies for the development of targeted therapeutics, such as immunostimulatory gene therapy, which can shift the tumor microenvironment toward an antitumor response.

Oncolytic virotherapy has received attention as a platform for targeted immunotherapy in part due to the FDA approval of Talimogene Laherparepvec (T-VEC or IMLYGIC® by Amgen, Thousand Oaks, CA, USA), a recombinant herpes simplex virus for late-stage melanoma [6,7]. Adenovirus is becoming increasingly recognized as a vector for immunotherapy due to several factors. With the approval of the first oncolytic adenovirus (ONYX-015) for human head and neck cancer, OAds have an extensively tested safety profile and have been shown to be well-tolerated in clinical trials [8]. In addition, adenoviruses have a high transduction efficacy for a variety of cancer cells and have a genome that can be manipulated with relative ease. They contain a large transgene cassette to allow for the expression of a number of desired molecules [9,10]. Furthermore, adenoviruses can infect and replicate in dividing and nondividing cells. However, they can be designed to specifically target cancer cells through selective infection or conditional replication after cell entry. The selectivity of infection has been explored using various methods, including capsid and fiber modifications [9,10]. Control of the replication of the vector can be achieved with a relevant promoter or mutation to the adenovirus early region genes. After replication of viral genes, production of viral proteins, and assembly of virus, cytolysis releases the oncolytic adenovirus (OAds) and allows for local spread of the vectors to neighboring cells, thereby producing an exponential antitumor effect.

The interactions between adenoviruses and the immune system can also be exploited to induce an antitumor effect. Cells of the innate immune system recognize pathogen-associated molecular patterns on the adenovirus. The production of type I interferons, IL-12 and GM-CSF (granulocyte macrophage colony-stimulating factor) increases, which results in activation of CD4+ and CD8+ T cells. Therefore, even if a small percentage of the cancer cells contains the target molecule for oncolytic adenoviral infection, a local pro-inflammatory response can be elicited to potentiate an antitumor response [4]. This response may be further enhanced when the OAds are armed with immunological effector molecules that counteract the immunosuppressive tumor milieu. In this review, we will first discuss several strategies that harness the power of OAds expressing immunostimulatory transgenes, such as GM-CSF, CD40L, interleukin-12, and interferon. In the second section, we will reflect upon some of the challenges that remain in the evaluation of OAds in the preclinical setting.

## 2. OAds (Oncolytic Adenoviruses) with Immunologic Effectors

The impetus for designing OAds to express immunostimulatory cytokines and chemokines is to increase the local concentration of these molecules in the tumor microenvironment, which would in turn enhance tumor-specific adaptive immune response. Consequently, two major strategies arise: Expression of a direct stimulator or antagonism of an inhibitor. In this first section, we will highlight OAds expressing immunostimulatory molecules, as well as CTLA-4 and combination therapeutic strategies that may further boost antitumoral immunity.

### 2.1. Granulocyte Macrophage Colony-Stimulating Factor (GM-CSF)

GM-CSF is a cytokine that is involved with the maturation of granulocytes, macrophages, and eosinophils. In addition, it causes the proliferation of vascular endothelial cells and activates dendritic cells and monocytes. Similarly, GM-CSF-based immunotherapy can activate monocytes and dendritic cells, thereby potentiating a T-cell based systemic antitumor response [11]. The use of oncolytic virotherapy for localized high-dose expression of GM-CSF in a tumor specific manner is increasingly recognized. T-VEC, a GM-CSF expressing oncolytic herpes virus, has been shown not only to be efficacious in advanced melanoma, but also can increase tumor antigen-specific T cells and decrease regulatory T cells and MDSC [7,12]. Advances in GM-CSF based virotherapies have led to the development of oncolytic adenoviruses for other types of solid tumors in recent years.

The Hemminki group has developed a GM-CSF-expressing OAd, which was first described in 2010 [13]. The Ad5/3-Δ24-GMCSF vector contains a chimeric fiber derived from the serotype 5 shaft and the serotype 3 knob [14]. The presence of a 24-basepair deletion [15] in the E1A region eliminates binding with the retinoblastoma protein (Rb), thereby allowing for selective replication in tumor cells with a deficiency in the p16-Rb pathway [13]. In vitro therapeutic effects using human cancer cell lines and in vivo antitumor effects in Syrian Golden Hamsters were demonstrated. In addition, the group has reported efficacy in human patients with metastatic solid malignancies who have failed treatment with conventional chemotherapy and radiation. Approximately half of these patients had either complete radiographic response, stable disease or minimal response when additionally treated with intratumoral injections of the Ad5/3-Δ24-GMCSF vector [13].

The group has also reported on the interactions between the recombinant OAd vectors and the immune system [16,17,18,19]. Antiviral antibodies against the Ad5/3 fiber and hexon were found to be elevated after only one treatment with OAds and remained elevated for several weeks after treatment [17]. Antitumoral response was assessed with interferon-γ Enzyme-Linked ImmunoSpot (ELISPOT) and the correlation between antitumor antibodies and clinical response. A positive correlation in the majority of patients has been observed between antitumoral response and induction of T cell response in two separate studies [18,19]. Furthermore, the majority of patients that experienced induction of antiviral responses also had antitumoral T cells; concordantly, those that did not exhibit antiviral response did not have antitumoral T cells [18].

A phase I study evaluating dose and safety of multiple intratumoral injections of the Ad5/3-Δ24-GMCSF vector in combination with cyclophosphamide has recently been published [20]. Now known as ONCOS-102, serial injections of the vector did not result in significant adverse events, despite a dose escalation design that allowed for the delivery of 3 × 10^10^ to 3 × 10^11^ viral particles per dose. Systemic pro-inflammatory cytokines including interleukin-6 and interleukin-8 did peak at 6 h post-injection, but decreased back to baseline values by 24 h post-injection. Histologic evaluation revealed that the majority of patients had increased CD8+ T cell infiltration, and IFN-γ ELISPOT showed response to tumor antigens. Clinical radiographic evaluation showed stable disease in 40% of patients [20].

Other groups have also attempted to exploit the Rb pathway in designing OAds expressing GM-CSF. In 2006, preclinical studies of CG0070, an E2F-1 promoter driven OAd armed with GM-CSF showed efficacy in both subcutaneous xenograft and orthotopic models of human bladder cancer [21]. E2F-1 is a transcription factor whose activation is suppressed by Rb [22,23]. With the E1A region of the OAds under the control of the transcription regulatory element E2F-1 promoter, the replication and cytotoxicity of CG0070, as well as the expression of its GM-CSF transgene, proceed selectively in tumor cells that are defective in Rb [21]. Serial intratumoral injections of CG0070 inhibited tumor growth, and histology demonstrated apoptosis within the tumor. While in vitro data demonstrated GM-CSF expression in human bladder cancer cell lines, local expression of GM-CSF from in vivo tumors was not shown. A non-randomized Phase I study to assess the safety of the CG0070 vector was published with 35 patients receiving either a single dose or multiple intravesical infusions [24]. After single dose treatment, half of the patients achieved complete response as evaluated by cystoscopy and serial biopsies. Most of the patients had only minor urinary adverse effect. The complete response rate increased to 60% after multiple doses. The concentration of GM-CSF in the urine and CG0070 viral DNA peaked only after the first infusion in the multiple-dose group [24].

Localized use of GM-CSF expressing OAd appears to be a promising therapeutic agent for the treatment of solid malignant tumors. While there is a paucity of randomized trial data in humans, the developed GM-CSF expressing OAds appear to be safe in preclinical trials. Further studies will be necessary to evaluate the efficacy of these vectors in a broader patient population.

### 2.2. CD40L

CD40 is a cell surface receptor that is present on all antigen presenting cells, hematopoietic progenitor cells, and activated B cells. The activity of the CD40 receptor and CD40L ligand pair is known to activate B cells and dendritic cells. Binding of the CD40L ligand (also known as CD154) onto B cells results in either cell growth in resting B cells or cell growth inhibition and immunoglobulin production in activated B cells. On antigen presenting cells, activation of CD40 enhances antigen presentation on dendritic cells and promotes Th1-oriented cell-mediated immunity [25]. For solid malignant carcinomas, CD40-CD40L interaction has been shown to directly inhibit proliferation and induce apoptosis [26,27]. In addition, binding of CD40L causes T cell expansion and increases in IL-12 production, which enhances cytotoxic T cell response to tumor antigens. Preclinical and early clinical trials with recombinant CD40L or CD40L gene transfer have demonstrated promising antitumor effect [25,26,27].

For oncolytic adenovirotherapy, CD40L has been attractive target in several settings due to the ability to limit CD40L expression to target sites for increased potency while reducing systemic toxicity. Taking advantage of CD40 overexpression in the majority of human breast cancers, the efficacy of an OAd expressing CD40L (AdEHCD40L) has been demonstrated [28]. The replication of this vector is driven by a hybrid promoter with an estrogen response element (ERE) and hypoxia response element (HRE). Specificity of the vector was demonstrated by enhanced cytotoxicity in CD40 positive breast cancer cells and decreased CD40L expression in nonmalignant epithelial cells. In addition, antitumor effect was seen after multiple intratumoral injections in SCID xenografts, along with increased apoptotic activity in the tumors injected with AdEHCD40L [28]. The vector also inhibited tumor growth in a subcutaneous xenograft model of multiple myeloma [29]. AdEHCD40L-treated multiple myeloma cells showed increased pro-inflammatory cytokine expression (such as IL-8 and RANTES (Regulated on Activation, Normal T Cell Expressed and Secreted)) at the mRNA and protein levels, compared to cells treated with the non-CD40L expressing AdEH vector [29].

More recently, a group in China evaluated the effect of a prostate-cancer targeted OAd expressing CD40L. The promoter for Ad-PL-PPT-E1A contains a chimera of androgen response element enhancer and prostate-specific antigen enhancer (PPT) and drives the expression of fusion gene of PSA and CD40L, PL [30]. The investigators showed selective oncolysis and replication in prostate cancer cells but not in tumor cells of other origins. Lysates from treated cells were able to stimulate dendritic cell maturation from human peripheral blood along with induction of cytokines expression such as IL-12, IL-6 and TNF-α. In vivo, multiple intratumoral injection of the vector resulted in tumor growth inhibition and localized production of CD40L within the tumor.

The Hemminki group has also developed a CD40L-expressing OAd vector that combines fiber modification and tumor-selective targeting [31]. The Ad5/3-hTERT-E1A-hCD40L (also known as CGTG-401) builds upon previously-developed replication selective adenovirus vectors driven by the human telomerase reverse transcriptase (hTERT) promoter [32,33,34]. CGTG-401 additionally contains the chimeric fiber from serotypes 5 and 3 for enhanced tumor transduction and encodes the human CD40 ligand [14,31]. In vitro, the investigators showed improved oncolysis in human CD40-positive endometrial cancer cells compared to CD40-negative lung cancer cells, as well as upregulation of markers of immunogenic cell death [31]. In vivo, syngeneic mouse models showed reduction in tumor growth and increased apoptosis after multiple intratumoral injections of a replication-deficient murine CD40L-expressing vector in immunocompetent mice. The increase in cytokines such as IL-12, RANTES, IFN-α and γ, as well as increased CD8 T cells in the treated tumors suggested an induction of cytotoxic T cell response. While the proof-of-principle was established in mice, the study was hindered by the inactivity of human CD40L in the murine system.

Therefore, a case report of the treatment of nine patients with the replication-competent CGTG-401 described the safety and assessed the immunologic response [35]. These patients had a variety of solid malignant tumors and received CGTG-401 intratumorally along with metronomic low-dose cyclophosphamide. No patients had any severe adverse side effects. Over half the patients had either stable disease or partial to complete response to adenoviral therapy. In select patients, induction of tumor-antigen stimulated CD8 cells was observed even one month after CGTG-401 injection and in half the patients, a Th1 immune dominant response was seen from the presence of IFN-γ, TNF-α and IL-2 cytokines [35]. Interestingly, patients treated with CGTG-401 had increased overall survival and progression-free survival, compared to historical controls. Therefore, CD40L-expressing OAd appears to be a promising modality for the treatment of metastatic solid tumors. However, the mechanisms by which it can induce an antitumor immune response is not yet fully elucidated and necessitates further detailed analysis.

### 2.3. Interferon

Interferons are a diverse group of cytokines with a wide range of biological functions and applications. Based on the receptor that they interact with, interferons can be categorized as Type I or II. In humans, there are six types of Type I interferons (IFN-α, IFN-β, IFN-ε, IFN-κ, IFN-ν, and IFN-ω), while Type II interferons consist only of IFN-γ [36]. Fourteen human IFN-α genes encode for twelve distinct proteins [36]. IFN-α has a multitude of effects including antiviral, antitumor, immunomodulatory, and antiangiogenic [37]. With these wide-ranging effects, IFN-α has been employed in numerous clinical trials for the treatment of a variety of solid tumors, as well as hematologic malignancies, such as hairy cell leukemia and chronic myeloid leukemia [38]. Nonetheless, systemic delivery of IFN-α is limited by dose-dependent toxicities. In addition, its short half-life limits its sustained availability as a drug. After intravenous administration, serum IFN-α concentrations peak within thirty minutes and becomes undetectable eight hours after injection [39].

Recent studies into the use of systemically-delivered IFN for the treatment of pancreatic cancer have highlighted both its tremendous therapeutic potential as well as its limiting side effects as a drug. Clinical trials have shown the effectiveness of chemoradiotherapy combined with IFN-α as part of an adjuvant treatment regimen. The Virginia Mason study group demonstrated that patients who received adjuvant therapy with IFN-α and 5-fluorouracil (5-FU) based chemoradiation had a significantly improved survival rate (84% at two years) compared to chemoradiation alone [40]. Long-term results of this study showed that the 10-year overall survival rate was 24% [41]. However, 70% of the treated patients developed grade 3 or higher systemic toxicities and over 40% of patients required hospitalization [41]. As a neoadjuvant therapeutic regimen, IFN-α and 5-FU based chemoradiation has shown some promise in converting unresectable or borderline resectable patients to resectable status for locally advanced pancreatic cancer. Patients who received surgical resection after neoadjuvant IFN-based chemoradiation had improved overall survival [42]. However, 30% of the patients in this study were not able to complete the regimen due to severe side effects [42].

Limitations of systemic IFN-α therapy have prompted investigators to use gene therapy approaches as a novel method to express/deliver IFN-α directly to pancreatic tumors. The Aoki group was the first to report the use of IFN-α gene transfer in pancreatic cancer [43]. They demonstrated that a replication defective adenovirus that locally delivered IFN-α limited the growth of and induced apoptosis in pancreatic cancer cells both in vitro and in vivo. Additional work by the same group analyzed the indirect and immunomodulatory effects of IFN-α delivery. They demonstrated that through direct intratumoral injection of an IFN-expressing adenovirus into subcutaneous xenograft tumors in nude mice, there was strong tumor suppression not only in the injected tumor but also in the uninjected contralateral tumor. The observed contralateral effect was mediated by systemic NK cell response [44]. Furthermore, a syngeneic immunocompetent hamster model demonstrated the induction of systemic immunity following intratumoral injection of an adenovirus that expressed IFN-α [45].

Our own research group has developed replication competent adenoviral vectors that express IFN-α for the treatment of pancreatic cancer. An infectivity enhanced, conditionally replicative OAd expressing IFN-α demonstrated selective replication in an ex vivo analysis using human tissue slices, as well as profound in vivo antitumor effects following intratumoral injections in a nude mouse model [46,47]. Additionally, treatment with a conditionally replicating IFN-expressing vector demonstrated significantly increased survival in a peritoneal carcinomatosis model in Syrian hamsters compared to untreated animals [48].

The application of adenovirus-based IFN-α therapy has not been limited to gastrointestinal malignancies. The Benedict group at the M.D. Anderson Cancer Center has investigated the use of intravesical delivery of IFN-expressing adenoviruses with Syn3 (a gene-transfer enhancing agent) for the treatment of bladder cancers. They demonstrated that the IFN-expressing vector resulted in prolonged expression of the IFN-α protein as well as significant antitumor activity [49]. This vector was then studied in a Phase I trial for patients with non-muscle invasive bladder cancer and was well tolerated [50]. IFN-α was detectable in patient’s urine up to 10 days following viral administration, demonstrating the efficiency of gene transfer [50]. Additionally, approximately 40% of patients experienced a complete response at three months, with some individuals remaining disease free for two to three years following intravesical adenoviral therapy [50]. Currently, this vector is undergoing a phase II investigation and a phase III study is planned for patients with non-muscle invasive bladder cancer (NCT02773849, clinicaltrials.gov).

Multiple other tumor types have been treated with adenovirus-mediated IFN-α delivery. Intracranial gliomas established in transgenic mice were treated with intratumoral delivery of an adenovirus vector expressing IFN-α and bone marrow-derived syngeneic dendritic cells. Here, half of the animals treated with this therapeutic regimen survived longer than 60 days, which was significantly longer than the survival observed in all other treatment and control groups [51]. Others diseases being investigated for the use of IFN-expressing adenoviral vectors include hepatocellular cancer, breast cancer, small cell lung cancer, and chronic myelogenous leukemia [52,53,54].

While systemic IFN-α therapy has shown promise, it is not well tolerated by patients. Adenovirus-based IFN-α delivery is one way to circumvent systemic side effects by allowing local delivery of high concentrations of the cytokine. It has been shown to be applicable for a wide variety of diseases and has the potential to be used in combination with other conventional treatment methods.

### 2.4. Interleukin-12

Interleukin-12 (IL-12) has received attention over the past 20 years due to extensive testing in preclinical trials and phase I human trials in advanced solid tumors [55]. In physiologic conditions, macrophages and dendritic cells secrete IL-12 after interacting with pathogens. IL-12 can activate NK cells and T cells, which eventually potentiate Th1 response and the induction of IFN-γ. In turn, IFN-γ enhances tumor antigen presentation via MHC-I complexes, as well as decrease angiogenesis and tumor invasion [56]. Treatment with recombinant IL-12 administered by various routes for solid tumors has produced disappointing response rates, fatality secondary to high systemic induction of IFN-γ, and low levels of IL-12 and IFN-γ within the targeted tumor site [57]. Therefore, OAds have been used to deliver high levels of IL-12 selectively at the tumor site in order to achieve the promised potent antitumor effect, while minimizing systemic inflammatory adverse effects.

In 2013, Freytag et al. developed an oncolytic adenovirus based on the group’s prior preclinical and phase I studies of OAd expressing two suicide genes [58]. The vector Ad5-yCD/mutTK_SR39_rep-mIL12 contains the cytosine deaminase/5-fluorocytosine and herpes simplex virus thymidine kinase/ganciclovir suicide genes, as well as the mouse IL-12 transgene. This vector also contains deletion of E1B 55-kDa, similar to that of ONYX-015, to allow for selective replication in p53-deficient cancer cells [59]. After showing cytotoxic effect in vitro, the investigators demonstrated longer survival and lower rate of metastatic disease, when vector administration was combined with 5-fluorocytosine and valganciclovir (a prodrug of ganciclovir) in a syngeneic model of mouse prostate cancer [58]. They further showed increased NK cell and cytotoxic T cell cytolytic activity within one week of intratumoral injections of the vector. Importantly, the significance of NK and cytotoxic T cell activity was highlighted in immune cell depletion studies, which demonstrated the mice experienced decreased survival and increased development of metastases with the depletion of NK, and CD4+ and CD8+ T cells [58].

The Yun lab from South Korea has developed multiple OAd vectors that express not only murine IL-12 but also other stimulatory molecules, such as B7-1, IL-18 and GM-CSF [60,61,62]. These OAds were modified from the background of the E1B 55 kDa deleted type 5 adenovirus. In 2006, the group demonstrated antitumor effect in vitro and in vivo with treatment of YKL-IL12/B7 in a syngeneic mouse melanoma model [62]. Interestingly, using a chromium release assay, the investigator demonstrated that mice treated with YKL-IL12/B7 and YKL-IL12 had greater cytotoxic T cell activity that was specific for tumor antigens, compared to the parental virus without IL-12 expression. Infiltration of CD4+ and CD8+ T cells and the presence of mature antigen presenting cells were demonstrated. Antitumor effect and induction of tumor-specific immune cells were also demonstrated with OAd expressing both IL-12 and IL-18 [60] and with OAd expressing IL-12 and GM-CSF [61].

In addition to immunocompetent syngeneic mouse models, OAd expressing murine IL-12 have been tested in syngeneic Syrian hamster models. In 2009, Bortolanza et al. developed Ad-DHscIL12, which selectively replicates in hypoxia, and tested the vector in intrahepatic lesions of hamster pancreatic cancer [63]. Accompanying the significant reduction in tumor volume was the increased expression of IFN-γ and IL-12, as well as survival advantage in the Ad-DHscIL12 group. In addition, stimulation of leukocyte proliferation was seen in the Ad-DHscIL12 group compared to the CMV promoter driver counterpart of the vector. Altogether, these results suggest that IL-12 expressing OAds have an antitumor effect and an ability to produce a tumor antigen-specific response in a replication-permissive immunocompetent model.

### 2.5. CTLA-4 Antibody

Contrary to the action of immunostimulatory molecules, binding of cytotoxic T lymphocyte-associated antigen-4 (CTLA-4) with B7 on antigen presenting cells acts to negatively regulate the activation of the T cell [4]. Blockage of CTLA-4 would allow for activation of tumor-specific T cells. The attractiveness of CTLA-4 as a target has been fueled by the development of two fully human monoclonal antibodies, tremelimumab and ipilimumab, which have gained FDA approval and have been incorporated into clinical guidelines for the treatment of advanced melanoma [64]. In addition, overexpression of CTLA-4 has been found in several malignant cancer cell lines including melanoma, colon cancer, breast cancer, neuroblastoma, and osteosarcoma [65], which produces an opportunity for targeted therapy. However, given the adverse effects that may accompany T cell activation from systemic administration, selective expression of CTLA-4 antibody using virotherapy is an intriguing therapeutic strategy.

The Hemminki group has developed an OAd expressing the complete human monoclonal CTLA-4 antibody [66]. Using the same modified backbone as the GM-CSF-expressing OAd, the Ad5/3-Δ24aCTLA4 vector also replicates selectively in tumor cells with a deficiency in the p16-Rb pathway. The biologic function of the CTLA-4 antibody expressed by the OAd was indirectly measured by the ability of infected cells to produce IL-2, a marker of T cell activation. The oncolytic activity of the vector was demonstrated in CTLA-4-positive ovarian, prostate and lung cancer cells, as well as human cancer xenografts in nude mice [66]. Additionally, four patients were treated to study the immunologic effect due to minimal activity of human CTLA-4 in mice. Increases in IL-2 and IFN-γ production after stimulation of peripheral blood from all four patients suggested increased T cell activity. This observation was not seen in two healthy donors [66]. How the recombinant CTLA-4 antibody compares to the CTLA-4 antibody expressing OAd in therapeutic effect remains to be seen.

### 2.6. Combination Therapy

While selective targeting of cancer cells with OAd as a monotherapy is appealing, many investigators are beginning to combine oncolytic viruses with other immunotherapies to overcome evasion of the immune system in the tumor microenvironment. In particular, PD-1 blockade with humanized antibodies, such as pembrolizumab and nivolumab has received great attention clinically due to its efficacy in advanced tumors [67]. Normally expressed on T cells, B cells and monocytes, PD-1 can bind to its ligand PDL-1 to inhibit T cell expansion. The rationale behind combination therapy is that while blockade of PD-1 binding would reduce inhibition of T cell activation, oncolytic viral burst would potentiate tumor antigen release, thereby synergistically inducing the adaptive immune system. Already, investigators have demonstrated efficacy in oncolytic viral platforms other than adenovirus. Recently, Rajani et al. explored the use of intravenous PD-1 antibody with intratumoral injections of oncolytic reovirus in a syngeneic mouse model of melanoma [68]. The investigators found increased survival in the mice that received the combination therapy, as well as increase IFN-γ, enrichment of NK cells and reduction of regulatory T cells [68]. In addition, oncolytic attenuated measles virus encoding single-chain variable regions of CTLA-4 and PD-L1 antibodies have been shown to delay tumor progression and increase survival in an immunocompetent syngeneic mouse model of melanoma [69]. Woller et al. showed therapeutic efficacy for the combination regimen of PD-1 immunotherapy and hTERT promoter-driven oncolytic adenovirus [70]. Oncolytic virotherapy reduced the burden of lung metastases in a syngeneic mouse model of lung cancer that appeared to be resistant to PD-1 immunotherapy. Additionally, the application of oncolytic adenovirus with PD-l blockade increases neoepitopes available for CD8 T-cell dependent antitumoral response [70]. Combining tumor selective OAd expressing immunostimulatory transgene with PD-1 antibody may further enhance tumor-specific immunity to overcome local immune tolerance, as well as potentiate immunologic memory to address metastatic disease.

Another method of overcoming evasion of immune surveillance is combining OAd with chimeric antigen receptor, CAR-T cells. The advantage of this method is the availability of the tumor-specific T cell that can produce the antitumor effect. The addition of OAds expressing immunostimulatory molecules would ensure the CAR-T cell reaches and remains at its intended target site. This therapeutic strategy was investigated by Nishio and colleagues, who combined RANTES and IL-15 expressing OAds with GD2-targeted CAR-T cells in an immunodeficient mouse model of neuroblastoma [71]. The local expression of RANTES and IL-15 by Ad5Δ24.RANTES.IL15 promoted the recruitment and persistence of GD2-targeted CAR-T cells, while causing cytotoxic effect on tumors and conferring longer survival [71]. This proof-of-principle of armed OAd enhancing the antitumor function of CAR-T cells shows an encouraging novel method by which an efficient antitumor response can be achieved.

## 3. OAds and the Immune System

While oncolytic adenovirus presents an exciting modality to eradicate solid tumors, several main questions regarding the interactions between the vector and the immune system remain. The mechanisms by which tumor suppression occurs and the involvement of the immune cells are complex and still being delineated. Does the immune response help or hamper the oncolytic effect of the adenoviral vectors? What type of an immune response do the OAd vectors elicit: Antiviral or antitumoral? These questions will be explored in this section.

Adenoviruses have been shown to interact with both the innate and adaptive immune systems. Through the activation of Toll-like receptors, viral infection causes dendritic cells to secrete cytokines, such as IL-6, IL-12, TNF-α and type I interferons [72,73]. Infected dendritic cells then affect the development of cytotoxic T cells by cross-presentation [74]. While OAds themselves can induce immune cells to secrete cytokines and chemokines, further arming oncolytic adenovirus with immunostimulatory transgenes ensures that a high local concentration of stimulatory molecule is readily present in the local tumor environment to influence immune effectors. This strategy addresses the limitations of systemic delivery of recombinant immunostimulatory molecules, which may cause an exaggerated cytokine response. Although 85% of population have neutralizing antibodies against human Ad5 [75], which may limit the efficacy of systemically delivered OAd, multiple methods of overcoming this hurdle is being investigated, including alterations to viral capsid proteins, usage of other serotypes of adenoviruses, and addition of synthetic polymers to the capsid, such as polyethylene glycol [76].

Although the secretion of proinflammatory cytokines to activate the immune system is one strategy to indirectly cause an antitumor effect, investigators have also sought to enhance the replicative efficacy of OAd, thereby improving direct oncolysis. Many of these studies focus on the use of systemic immunosuppressants, such as cyclophosphamide or temozolomide. In a series of studies, Wold and his group evaluated the impact of cyclophosphamide on the OAd VRX-007, adenovirus type 5 that overexpress the adenovirus death protein [77]. With the administration of cyclophosphamide given prior to OAd infection in a syngeneic model of renal cancer in the Syrian hamster, immunosuppression resulted in a significant reduction in tumor volume, compared to the group without immunosuppression. The immunosuppression also resulted in a consistently elevated level of OAd in the tumors, as well as liver and blood, but did not result in significant histologic hepatotoxicity over the course of observation [77]. The antitumor effect was observed even in the presence of neutralizing antibodies [78] and with a shorter course of cyclophosphamide treatment [78,79]. The sustained oncolysis and elevated intratumoral OAd levels observed with cyclophosphamide may be a promising therapeutic regimen. However, the side effects of cyclophosphamide are not minimal. While local oncolysis can elicit a systemic T cell response, the effect of the combination therapy with cyclophosphamide and OAd on the rise of future metastatic diseases is yet to be fully evaluated.

Another point of debate lies in whether tumor-selective OAds elicits an antiviral or antitumoral response, or both. For induction of antiviral immunity, activation of dendritic cells starts with recognition of viral DNA through pathogen-associated molecular patterns. Studies in immunogenicity of adenovirus-based vaccines have demonstrated that induction of viral antigen specific CD8+ T cells occur via MyD88-dependent Toll-Like receptor signaling [80]. In the case of adenovirus-based oncolytic virotherapy, the selective expression of viral protein and viral DNA replication in targeted cancer cells would elicit not only innate response but also an antiviral T cell response and cross-priming to the adaptive immune system from cellular antigens [74,81]. An antitumoral response through activation of the adaptive immune system can enhance the destruction of the primary tumor as well as metastatic disease, as a result of oncolytic burst and release of tumor antigens.

Kubicka and his group have attempted to dissect out the dichotomy of antiviral versus antitumoral response elicited from the use of OAds [82,83]. The group has described the use of modified OAd type 5 whose replication is driven by hTERT promoter and is reliant upon the presence of a dysfunctional p53 (Ad-p53T) in a syngeneic immunocompetent mouse model [83]. The investigators demonstrated that intratumoral injections of the selective OAd resulted in reduced induction of antiviral adaptive CD8+ response, compared to the nonselective OAd. In addition, analysis of nontargeted tissue such as the liver revealed significantly reduced toxicity with use of selective OAd. When the selective OAd was administered to subcutaneous tumors in a metastatic tumor model, the growth of the uninjected lung metastases was inhibited and the survival of the animals increased, suggesting induction of systemic tumor specific T cells [83].

## 4. Concluding Comments

With greater understanding of how adenovirus interacts with various immune cells of the tumor microenvironment, OAds have become an increasingly appealing and easily modifiable platform that can be selectively targeted to tumor cells. Expression of immunologic effector molecules, such as GM-CSF, IL-12, CD40L, and IFN-α allows OAds to become chaperones of these key pro-inflammatory cytokines and chemokines, which accumulate in a selectively targeted area within the tumor. As hitherto described in the preceding sections, many investigators have developed innovative OAds expressing immunostimulatory molecules that harness tumor immunity to produce a greater antitumor response. While there have been several promising OAds with immune-enhancing function in preclinical models, the bench-to-bedside transition has been deterred by the absence of an adequate animal model that would permit both human adenovirus replication and detailed immunologic analyses for predicting immunological effects in patients. Investigations into alternative animal models and well-controlled clinical trials may need to be pursued concomitantly in order to fully assess if OAds carrying immunostimulatory molecules can be safely delivered to humans. Additional clarification regarding the mechanism by which OAds exert their antitumor effect and influence the cross-talk between immune and cancer cells would enable investigators to design more efficiently targeted vectors that would elevate them to a closer clinical reality.
